# 2,4-Di-Tert-Butylphenol Isolated From an Endophytic Fungus, *Daldinia eschscholtzii*, Reduces Virulence and Quorum Sensing in *Pseudomonas aeruginosa*

**DOI:** 10.3389/fmicb.2020.01668

**Published:** 2020-07-27

**Authors:** Rashmi Mishra, Jai Shanti Kushveer, Mohd. Imran K. Khan, Sudhakar Pagal, Chetan Kumar Meena, Ayaluru Murali, Arunkumar Dhayalan, Vemuri Venkateswara Sarma

**Affiliations:** ^1^Department of Biotechnology, Pondicherry University, Puducherry, India; ^2^Centre for Bioinformatics, Pondicherry University, Puducherry, India

**Keywords:** anti-quorum sensing, 2, 4-Di-tert-butylphenol, endophytic fungi, multidrug resistance, *P. aeruginosa*

## Abstract

*Pseudomonas aeruginosa* is among the top three gram-negative bacteria according to the WHO’s critical priority list of pathogens against which newer antibiotics are urgently needed and considered a global threat due to multiple drug resistance. This situation demands unconventional antimicrobial strategies such as the inhibition of quorum sensing to alleviate the manifestation of classical resistance mechanisms. Here, we report that 2,4-di-tert-butylphenol (2,4-DBP), isolated from an endophytic fungus, *Daldinia eschscholtzii*, inhibits the quorum-sensing properties of *P. aeruginosa.* We have found that treating *P. aeruginosa* with 2,4-DBP substantially reduced the secretion of virulence factors as well as biofilm, and its associated factors that are controlled by quorum sensing, in a dose-dependent manner. Concomitantly, 2,4-DBP also significantly reduced the expression of quorum sensing-related genes, i.e., *lasI*, *lasR*, *rhlI*, and *rhlR* significantly. Importantly, 2,4-DBP restricted the adhesion and invasion of *P. aeruginosa* to the A549 lung alveolar carcinoma cells. In addition, bactericidal assay with 2,4-DBP exhibited synergism with ampicillin to kill *P. aeruginosa.* Furthermore, our computational studies predicted that 2,4-DBP could bind to the *P. aeruginosa* quorum-sensing receptors LasR and RhlR. Collectively, these data suggest that 2,4-DBP can be exploited as a standalone drug or in combination with antibiotic(s) as an anti-virulence and anti-biofilm agent to combat the multidrug resistant *P. aeruginosa* infection.

## Introduction

Quorum sensing is a bacterial signaling mechanism through which bacteria sense their cell density and activate a range of coordinated behaviors once their population reaches a threshold (Rutherford and Bassler, 2012). Bacteria release signaling molecules, called autoinducers, which accumulate as the cell density of the bacteria increases. QS regulates an array of bacterium physiological activities, such as virulence, pathogenesis, biofilm formation, swimming, and swarming motility. Since several of these functions are central to bacterial persistence and pathogenesis, QS has been regarded as an attractive target for anti-biofilm and anti-QS-based alternative anti-microbial therapy. However, little progress has been made concerning QS-based alternative anti-microbial therapies. *Pseudomonas aeruginosa*, well known to be an opportunistic, notorious nosocomial pathogen responsible for causing a range of acute and chronic infections, such as respiratory tract infections, urinary tract infections, infections in the central nervous system, and skin and soft tissue infections in immuno-compromised patients (Bjarnsholt et al., 2010). *P. aeruginosa* engages in QS by three independent, but by cross-talking, LasR-LasI, RhlR-RhlI, and PQS-PqsR QS signaling systems, where the autoinducer for the LasR-LasI system is N-(3-oxo-dodecanoyl) homoserine lactone (3OC_12_ HSL), and the RhlR-RhlI system utilizes C4 (butanoyl) HSL (Pearson et al., 1995). The LasRI system of this bacterium regulates the expression of several genes encoding various virulence factors (Schuster et al., 2003). The autoinducer of the third QS system PQS-PqsR, 2-heptyl-3-hydroxy-4(1H) quinolone (PQS), binds to the transcriptional regulator PqsR and further controls downstream targets, including biofilm formation, which leads to antibiotic tolerance and resistance without the need for specific antibiotic inactivating enzymes. Therefore, QS inhibitors could have this dual advance of rendering the bacterium non-virulent and sensitizes it toward antibiotics. QS inhibitors are anticipated to curtail the pathogenicity, since the expression of several virulence factors and the facilitation of a successful infection are under QS regulation (Pearson et al., 2000). Hence, molecules interfering QS could be an aid to the existing armamentarium against *P. aeruginosa* infections. These strategies include degrading AHL molecules enzymatically by acylases, lactonases, and oxidoreductases, outcompeting/inhibiting QS signal molecules by structurally similar inhibitory molecules to bind to their cognate regulatory proteins, or by quorum quenching antibodies and macromolecules such as cyclodextrins that scavenge autoinducers (Rémy et al., 2018; Ahmed et al., 2019). In addition, several natural substances with known biological properties act as QS inhibitors as they intervene in QS-associated pathways, attenuate QS gene expression, and impair the infection. Recently, several reports claim to quench QS or ameliorate the QS signals through various synthetic molecules, natural products, and enzymes (Fong et al., 2018). For instance, acyl homoserine lactone analogs such as N-acyl cyclopentyl amines (Cn-CPAs), lactonase SsoPox, N-acylhomoserine lactonase, and AiiM (Guendouze et al., 2017; López-Jácome et al., 2019) were effective as QSIs against *P. aeruginosa.* Curcumin and coumarin were reported to inhibit the virulence and biofilm-forming ability of *P. aeruginosa*, while naringenin and taxifolin were reported to reduce the expression of QS-related genes. Furthermore, enzymes such as AHL-lactonases are reported to degrade 3OC8HSL of *P. aeruginosa* and affect the virulence capability and biofilm-forming ability (Kalia et al., 2018).

Recently, we reported that metabolites from endophytic fungi associated with *Carica papaya* also attenuate in *P. aeruginosa* (Mishra et al., 2018; Meena et al., 2019). Besides being ecologically and physiologically diverse, endophytic fungi are diverse in synthesizing chemically potent and varying secondary metabolites when in association with a medicinally important host (Rashmi et al., 2019). In the present study, we report 2,4-DBP as a QS inhibitor that was isolated from the endophytic fungi *Daldinia eschscholtzii* associated with host plant *Tridax procumbens*, which is known for its traditional medicinal values (Mir et al., 2017). 2,4-DBP not only impeded QS-mediated virulent factors and biofilm formation but also showed synergistic effects with therapeutically relevant antibiotics. Finally, *in silico* analyses showed it to be an effective QS inhibitor and comparable to the known anti-QS inhibitor BAC.

## Materials and Methods

### Organisms and Reagents

*Chromobacterium violaceum* ATCC 12472 and *Pseudomonas aeruginosa* PAO1 are the test strains used in the study. Cultures were maintained in Luria–Bertani (LB) broth and routinely subcultured. BAC standard (Sigma-Aldrich, United States) was dissolved in dimethyl sulfoxide (Merck) and was sterilized using a 0.22-μm PVDF membrane filter. Chitin azure, azocasein, and elastin congo red were procured from Sigma-Aldrich (Sigma-Aldrich, United States), Maxima H Minus Reverse Transcriptase from Thermo Scientific, and FastStart Universal SYBR Green Master Mix from Roche (USA). The A549 lung epithelial cell carcinoma cell line was procured from the National Center for Cell Sciences, India, for *in vitro* infection studies.

### Isolation and Screening of Potential Endophytic Fungi

Green and healthy leaves of the host *Tridax procumbens* were collected from the Pondicherry University campus, India, 12.0219° N, 79.8575° E. After surface sterilization according to Cui et al. (2015), endophytic fungi were isolated, subcultured, and maintained as axenic cultures. All the endophytic fungi isolated were screened for their anti-QS potential against *Chromobacterium violaceum* (ATCC 12472) and *Pseudomonas aeruginosa* PAO1. Agar well diffusion method was performed to treat the fungal crude samples to a lawn of bacteria and examine the zone of inhibition. The isolate with the largest zones of inhibition was considered as most effective and selected for the rest of the work. The most potent isolate was further subjected to purification of the QS inhibitor compound as described below.

### Identification and Phylogenetic Analysis of Selected Endophytic Fungi

After screening the isolates, isolate TP2-6 (an in-house code) was selected for further study. The morphological details of the fungi were observed under stereo-zoom microscope and compound microscope for various characteristics of colony and spore formation (Mishra et al., 2018). For molecular identification, the nuclear ribosomal internal transcribed spacer (ITS) region was amplified by the primers ITS1 (5′-TCC GTA GGT GAA CCT GCG G-3′) and ITS 4 (5′-TCC TCC GCT TAT TGA TAT GC-3′) and sequenced by capillary sequencing, followed by phylogenetic analysis as described in Devadatha et al. (2018).

### Large-Scale Fermentation of *Daldinia eschscholtzii* and Extraction of Crude Extract

The endophytic fungal isolate *Daldinia eschscholtzii* (TP2-6), which showed the most potent anti-QS activity, was selected for the purification of the active compound responsible for the anti-QS activity against *P. aeruginosa*. About 20 liters of potato dextrose broth (PDB) was used to cultivate *D. eschscholtzii*. From the axenic culture of *D. eschscholtzii*, a loop full inoculum was inoculated into PDB and it was kept for growth for 20 days at 28°C at constant agitation. The fungal broth, after separating the mycelium, was extracted twice with double the volume of ethyl acetate. The organic phase was separated and vacuum dried to obtain the fungal crude extract.

### Column Chromatography

The concentrated crude sample of *D. eschscholtzii* was further subjected to column chromatography for purification in a glass column (700 × 30 mm). The glass column was packed with silica gel (60–120 mesh size, Merck) as the stationary phase. A dried powdered crude extract mixed with silica gel/powder (200 mesh size) at a ratio of 1:3 was loaded onto a column as the sample bed. The column was first eluted with hexane, followed by a hexane: ethyl acetate mixture with a gradual increase in polarity in different ratios (9:1, 8:2, 7:3, 6:4, 5:5, etc.), and finally eluted by using 100% of ethyl acetate, methanol, and water. Different fractions were collected and assessed for their anti-QS activity.

### High-Performance Liquid Chromatography (HPLC)

High-performance liquid chromatography of the active fraction was done on an RP-C18 column using photodiode array detectors (PDA-SPD-M20A). The injection volume and flow rate used were 10 μL and 0.50 mL/min, respectively. Acetonitrile along with HPLC-grade water was used as the mobile phase solvent. The elution program of compounds started with 15% acetonitrile reaching up to 100% in 40 min with a hold on this condition for 5 min, and again gradient coming down to 15% acetonitrile in 8 min which was finally held for 5 min (Sharma et al., 2017). The samples and mobile phase were filtered through a 0.2-μm nylon membrane filter before applying into the column. Samples were analyzed at 280 nm wavelength.

### Characterization and Structure Analysis

Fourier transform infrared spectroscopy (FTIR) of the isolated compound was performed with a Thermo Nicolet model 6700 IR source range from 500 to 4000 cm^–1^ to obtain an IR spectrum to analyze the functional group present in the compound. High-resolution mass spectroscopy (HRMS) was used to determine the molecular mass of the compound using Agilent 6530B, Agilent mass Q-TOF LC/MS. The structure of the isolated pure compound was determined with the help of nuclear magnetic resonance (NMR) spectroscopy using a Bruker Avance II 400 spectrometer (US).

### Anti-QS Potential of the Isolated Pure Compound

The compound isolated from the fungal extract (2,4-DBP, a known compound) was further investigated for its anti-QS and anti-biofilm activity against *P. aeruginosa* as described below. To compare, BAC, a well-known phyto-compound known for its ability to attenuate the virulence factors of *P. aeruginosa* by downregulating the transcription of QS-regulated genes, was used as positive control. Dimethyl sulfoxide (DMSO) was used as a negative control. *P. aeruginosa* was grown in LB broth to attain an OD_600_ of 0.4. It was further incubated in the presence of 2,4-DBP or BAC for 18 hrs. To obtain a cell-free culture supernatant for different assays, bacterial cells were pelleted down by centrifugation at 10,000 rpm for 10 min.

#### Determination of Sub-MIC and Growth Curve Analysis

Microbroth dilution, using the Clinical and Laboratory Standards Institute (CLSI) standard method as described in Luo et al. (2016), was used to determine the MIC of 2,4-DBP and BAC against *P. aeruginosa*. Consequently, sub-MICs were selected to perform further experiments.

#### Effect of 2,4-DBP on the Production of Virulence Factors

##### Violacein production assay

A visual investigation of the ability of 2,4-DBP to attenuate the QS-regulated violacein pigment production in *C. violaceum* was performed using agar well diffusion assay as described in Rajkumari et al. (2018a). A quantitative estimation of the inhibition of violacein production by *C. violaceum* was performed when treated with 2,4-DBP and BAC at respective sub-MICs by spectrophotometric measurement at 585 nm of the supernatant (Rajkumari et al., 2018a).

##### Pyocyanin production assay

A quantitative chemical assay was used to measure the inhibition of pyocyanin pigment production. Briefly, 1 mL of cell-free culture supernatant of *P. aeruginosa* grown with 2,4-DBP and BAC at appropriate concentrations was extracted with an equal volume of chloroform. After extraction, the organic phase was extracted by 1 mL of 0.2 N HCl, and the amount of pyocyanin was estimated spectrophotometrically at 520 nm (Ganesh and Rai, 2016).

#### Proteolytic Activity Assay

*Pseudomonas aeruginosa* secretes several proteases that serve as key mediators to establish an acute infection.

##### Chitinase activity assay

Modified chitin azure assay was used to determine the inhibition in chitinase activity (Husain et al., 2013). Chitin azure (0.5 mg/mL) dissolved in sodium citrate buffer (0.1 M, pH 4.8) was used a substrate. Concisely, 1 mL of the cell-free supernatant of *P. aeruginosa* was mixed with 0.5 mL of substrate solution, and the mixture was incubated for 7 days at 37°C in constant agitation (150 rpm). After removal of the insoluble substrate by centrifugation at 10,000 rpm, absorbance of the collected supernatant was recorded at 570 nm.

##### LasA staphylolytic assay

LasA staphylolytic activity, an ability to lyse heat killed cells of *Staphylococcus aureus*, was estimated as described by Kessler et al. (1993). Harvested pellet of *S. aureus* cells grown overnight was resuspended in 0.02 M Tris (pH 8.5) to obtain an OD_600_ of 0.8. 100 μL of cell-free supernatant of *P. aeruginosa* (obtained as described above) was mixed with 900 μL of *S. aureus* suspension. After an incubation of 1 h, the cell density was measured at 600 nm.

##### LasA protease assay

Proteolytic activity of *P. aeruginosa* was estimated as reported by Hentzer et al. (2002) with some modifications. Briefly, 500 μL of substrate solution and 0.3% azocasein [prepared in 50 mM Tris (pH 7.8)] were mixed with 100 μL of cell-free supernatant of *P. aeruginosa* for 30 min at 37°C. Finally, 0.5 mL of prechilled 10% trichloroacetic acid was added and incubated for 15 min at 4°C to precipitate the undigested substrate. The protease activity was recorded as absorbance at 400 nm of the clear supernatant obtained after centrifugation at 10,000 rpm.

##### LasB elastase assay

The elastolytic activity of the cell-free supernatant of *P. aeruginosa* was measured according to Ohman et al. (1980). In brief, 100 μL of culture supernatant of *P. aeruginosa* was added to 900 μL of elastin congo red buffer (100 mM Tris, 1 mM CaCl_2_, pH 7.5) containing 20 mg of elastin congo red. The reaction mixture was incubated at 37°C for 3 h. Finally, the elastolytic activity was recorded as absorbance at 495 nm of the clear supernatant obtained after centrifugation at 10,000 rpm for 10 min.

#### Motility Assay

The effect of sub-MIC concentrations of 2,4-DBP and BAC on the motility, i.e., swimming and swarming ability of *P. aeruginosa*, has been investigated as described in Mishra et al. (2018). Treatment with BAC acted as a positive control whereas the untreated sample acted as internal control and 2,4-DBP acted as treatment.

#### Hydrogen Cyanide (HCN) Production Assay

The production of HCN by *P. aeruginosa* as one of its virulent factors was assayed according to Reetha et al. (2014). King’s B medium agar plates supplemented with glycine were prepared with and without test compounds. After streaking *P. aeruginosa* onto the plates, a filter paper saturated with 0.5% picric acid, fortified with 2% of Na_2_CO_3_, was placed on the roof of the lid of the petri dish. The plates were tightly sealed and incubated for 24 h at 37°C. Production of HCN caused a change of color from yellow to orange.

#### Effect of 2,4-DBP on Biofilm Formation and Associated Factors of *P. aeruginosa*

##### Microtiter plate biofilm assay

The inhibitory effect of 2,4-DBP on the biofilm formation by *P. aeruginosa* was investigated according to Luo et al. (2016). *P. aeruginosa* was grown in 96-well flat-bottomed microtiter plates in the presence and absence of 2,4-DBP and BAC for 24 h at 37°C. After incubation, the wells are washed with sterile phosphate-buffered saline (PBS) to remove unadhered cells. The biofilm was stained with 1% crystal violet for 5 min and again washed with sterile PBS to remove excess stain. The crystal violet stained biofilm was dissolved with 33% acetic acid and was quantified by absorbance at 595 nm.

##### Extraction and quantification of exopolysaccharides (EPS)

The secreted exopolysaccharide (EPS) was quantified as reported by Packiavathy et al. (2014). The cell-free culture supernatant of *P. aeruginosa* was precipitated by three volumes of chilled ethanol (100%). It was incubated for 24 h at 4°C. The precipitated EPS was pelleted by centrifugation (10000 rpm, 15 min) and dissolved in Milli-Q water. EPS was quantified using the phenol-sulfuric acid method, wherein 1 mL of 5% cold phenol and 5 mL of conc. H_2_SO_4_ were mixed with 1 mL of EPS suspension, which was quantified spectrophotometrically 490 nm.

##### Extraction and quantification of rhamnolipids

Rhamnolipid extraction and quantification were performed as reported by Luo et al. (2017). *P. aeruginosa* was grown with and without 2,4-DBP and BAC (as described above), and 1 mL of cell-free culture supernatant (obtained as described above) was extracted with twice the volume of ethyl acetate and dried. The dried extract was resuspended in 900 μL of orcinol solution (0.19% orcinol dissolved in 53% v/v sulfuric acid). The mixture was incubated for 30 min at 80°C and quantified at 421 nm spectrophotometrically (Luo et al., 2017).

##### Extraction and quantification of alginate

The alginate extraction and quantification from the cell-free culture supernatant of *P. aeruginosa* were performed as described by Rashmi et al. (2018). Concisely, 0.6 mL of boric acid–sulfuric acid (4:1) solution was mixed with 70 μL of cell-free supernatant and vigorously mixed on an ice bath for 10 s. About 20 μL of carbazole solution (0.2% carbazole dissolved in ethanol) was added to the previous mixture, followed by centrifugation (10,000 rpm, 10 min). This was followed by incubation at 55°C for 30 min. Alginate was measured by absorbance at 530 nm.

##### Cell-surface hydrophobicity (CSH) assay

The methodology for estimating cell-surface hydrophobicity was employed as earlier reported by Viszwapriya et al. (2016) with minor modifications. Briefly, 1 mL of *P. aeruginosa* culture was cultivated with and without 2,4-DBP and BAC and mixed with 1 mL of toluene with vigorous vortexing for 2 min. The aqueous phase was collected for bacterial cell density measurement at 600 nm. The CSH indicated by the ability of cells to adhere to the hydrophobic substrate (here, toluene) was calculated as CSH% = [1 - (OD_600_ after vortexing/OD_600_ before vortexing)] × 100.

##### Congo red agar biofilm formation assay

The Congo red agar method was performed as reported by Kuzu et al. (2012). Congo red dye (0.08%) was added to the agar medium containing comprised brain heart infusion broth (BHI-37 gm/L), agar (1%), and sucrose (0.5%). Media plated were prepared with and without 2,4-DBP and BAC. *P. aeruginosa* was streaked on the congo red plates and incubated for 24–48 h at 37°C. The presence of dry crystalline black colonies confirmed the exopolysaccharide (EPS) production.

##### Extracellular DNA (eDNA) quantification

The supernatant of *P. aeruginosa* was filter sterilized through a 0.22-μm membrane and treated with an equal amount of phenol/chloroform/isoamyl alcohol (25:24:1), and the mixture was vortexed for a few seconds. The eDNA in the supernatant (500 μL) was then precipitated by sodium acetate (200 μL) and ice-cold isopropanol (1.3 ml). The precipitated eDNA was pelleted by centrifuging it at 12,000 × *g* for 15 min at 4°C. The pellet obtained was resuspended in 40 μL of TE buffer (1 mM EDTA and 10 mM Tris, pH 8.0). The eDNA suspension was treated with 10 μL proteinase K (10 μg/μL) followed by incubation at 37°C for 1 hr. With the help of NanoDrop Fluorospectrometer, the eDNA was quantified and electrophoresed in agarose gel (0.8% [w/v] agarose in TBE buffer) and visualized in the gel documentation system.

##### Microscopic analysis of biofilm

The biofilm formation on the abiotic surface was assayed as described by Rajkumari et al. (2018a). In a 24-well microtiter well plate containing Tryptic Soy Broth and coverslips (1 × 1 cm), 1/100th diluted overnight *P. aeruginosa* broth culture was grown for 24 h at 37°C. For light microscopic analysis, the coverslips were washed with sterile PBS to remove unadhered cells and stained with 0.4% crystal violet for 10 min. For fluorescence microscope analysis, the coverslips were stained with acridine orange (4 μg/mL) in the dark and then washed with PBS to remove excess stain. The coverslips were allowed to dry and visualized under respective microscopes.

### RNA Isolation and Quantitative Real-Time PCR (qRT-PCR)

The total RNA was extracted from Pseudomonas aeruginosa cultures grown for 18 h in the presence of the test compounds. The cultures were grown for 18 hrs to allow maximum exposure of the test compounds to the bacterium. The RNA isolation and cDNA synthesis were performed as described in Mahesh et al. (2020). The primers that were used in qRT-PCR are listed in Supplementary Table S2. Data were analyzed by the ΔΔCt method. Each qRT-PCR reaction was performed in triplicates, and the assays were repeated thrice. Data were normalized to the housekeeping gene *rpoD* expression.

### *In silico* Studies

Three-dimensional structures of ligands were docked to three-dimensional structures of proteins to check their binding affinity. This was followed by molecular dynamic simulations to get an insight into the effect of this binding on the three-dimensional structure of the proteins and the stability of the complex.

#### Docking

Molecular docking of LasR and RhlR with 2,4-DBP (which is isolated from *D. eschscholtzii* crude extract) was performed to evaluate their interaction strengths in comparison to their cognate ligands and BAC, using the “induced fit docking” module of Schrodinger (Schrodinger Inc., United States). These molecules were retrieved from PubChem, with the IDs as given in Supplementary Table S3. Protein was prepared through “protein preparation wizard” of the Schrodinger docking suite 2018. Ligands were prepared using the “ligprep” module.

#### Molecular Dynamic Simulation

Protein and protein–ligand complexes were simulated by Gromacs 5.1.4 simulation package using the “gromos” force field (Abraham et al., 2015). All the complexes were placed into a cubic box of size 2 Å along with the SPCE water model as the solvent. The system was equilibrated well before final simulation of 20 ns with the time step of 10 ps.

### *In vitro* Infection Studies

The A549 lung epithelial carcinoma cells were infected with *P. aeruginosa* PAO1 in the presence of the identified compounds to evaluate if they interfere with host-cell infection by the bacterium. Host cells were grown in DMEM containing 10% FBS (fetal bovine serum) and L-glutamine–penicillin–streptomycin (0.5%) solution at 37°C in 5% CO_2_ condition.

#### Adhesion Assay

The extent of (host) cell adhesion was evaluated by the procedure described in Hawdon et al. (2010). Confluent A549 cells were incubated with *P. aeruginosa* with a multiplicity of infection (MOI) of 100 (resuspended with DMEM) in the presence or absence of BAC (120 μg/mL) or 2,4-DBP (80 μg/mL) and incubated at 37°C for 1 h to allow bacterial adhesion. Wells with bacteria but no test compound served as positive control while the uninfected wells served as negative control. Uninfected host cells with BAC or 2,4-DBP were also kept. The wells were washed thrice with sterile PBS to remove non-adhered bacteria, followed by trypsinization with least possible trypsin lysed with 70 μL of 0.1% Triton X-100 (Sigma) at room temperature. The lysed cells were collected, serially diluted, and plated onto the LB agar plate for counting colony-forming units (CFU).

#### Invasion Assay

To enumerate the extent of host-cell invasion by *P. aeruginosa*, A549 cell invasion assay was performed according to Hawdon et al. (2010). Confluent A549 cells were infected with *P. aeruginosa* (resuspended with DMEM) at MOI of 100, in the presence or absence of BAC (120 μg/mL) or 2,4-DBP (80 μg/mL). The plates were incubated at 37°C for 2 h to allow internalization of the bacterial cells. Wells were washed with sterile PBS and then incubated with fresh DMEM supplemented with gentamicin (200 μg/mL) for 1 h to kill extracellular bacteria. Following incubation, the cells were washed thrice with sterile PBS, trypsinized, and lysed with 70 μL of Triton X-100 (0.1%). The suspension was serially diluted and plated onto LB agar for CFU count. Host cells with bacteria but no test compound served as a positive control, while uninfected cells served as negative control. Uninfected host cells with BAC or 2,4-DBP were also kept. All cocultures were performed in triplicates.

#### Live/Dead Cell Imaging by Acridine Orange (AO)/Ethidium Bromide (EB) Staining

A549 cells were grown to about 90% confluence in a 35-mm cell-culture dish and infected with *P. aeruginosa* at MOI of 100 in the presence or absence of 2,4-DBP (80 μg/mL) and BAC (120 μg/mL). After 24 hrs of incubation, the medium was discarded, followed by washing of the cells thrice with sterile PBS. Finally, 20 μL of the AO/EB mix (4 μg/mL) was used to stain the cells and viewed under a fluorescent microscope with a B-2A filter (Nikon Eclipse TS100, Japan). EB stains only dead cells, whose membranes are permeable, whereas AO stains all cells. Hence, dead cells fluoresce red-orange while live cells fluoresce green.

### Synergistic Antimicrobial Studies of 2,4-DBP With Antibiotics

*Pseudomonas aeruginosa* PAO1 was screened against therapeutically relevant antibiotics, which showed that the strain is resistant to ampicillin. Its MIC against the strain was determined by micro-broth dilution method as per CLSI guidelines. Following this, various combinations of ampicillin and 2,4-DBP (combinations) were used against *P. aeruginosa* to investigate any potential synergistic efficacy in inhibiting that bacterium. An overnight grown culture of *P. aeruginosa* was diluted to 1/100th, 200 μL of which was dispensed into the wells of 96-well microtiter plates and treated with the 2,4-DBP-ampicillin combinations. Untreated wells served as controls. Each treatment was performed as triplicates. After 24 h of incubation, the growth of the cells were monitored and expressed the cell viability in terms of percent CFU.

### Mathematical Calculations and Statistical Analysis

Each experiment was performed in triplicates, and values were expressed as standard means with standard deviations. Values for 2,4-DBP-treated experiments are normalized with those BAC-treated whenever appropriate. All the cultures were adjusted to a set OD of 0.4 at 600 nm before the experiments.

The percentage of inhibition in different assays was calculated as follows:

$$\mathrm{Percentage}\,\mathrm{inhibition}=\left(1-\frac{\mathrm{Ab}.\left(\mathrm{sample}\right)}{\mathrm{Ab}.\left(\mathrm{control}\right)}\right)*100$$

where Ab. (control) = Absorbance of control, Ab. (sample) = Absorbance of treated sample, at respective wavelengths.

Statistical analyses of all the experiments were performed in Microsoft Excel MegaStat software. Data readings of all experiments were documented as mean ± standard deviation. The *p*-values < 0.05 represent the significance of the conclusion.

## Results and Discussion

### Endophytic Fungus *Daldinia eschscholtzii* Shows Anti-QS Activity

A total of 32 endophytic fungi were isolated from the leaves of *Tridax procumbens*. Their extracts were screened against *P. aeruginosa* and *C. violaceum* for anti-QS activity. Out of them, the isolate with code TP2-6 showed the best activity and hence was selected for further studies (Supplementary Table S1).

**FIGURE 1:**
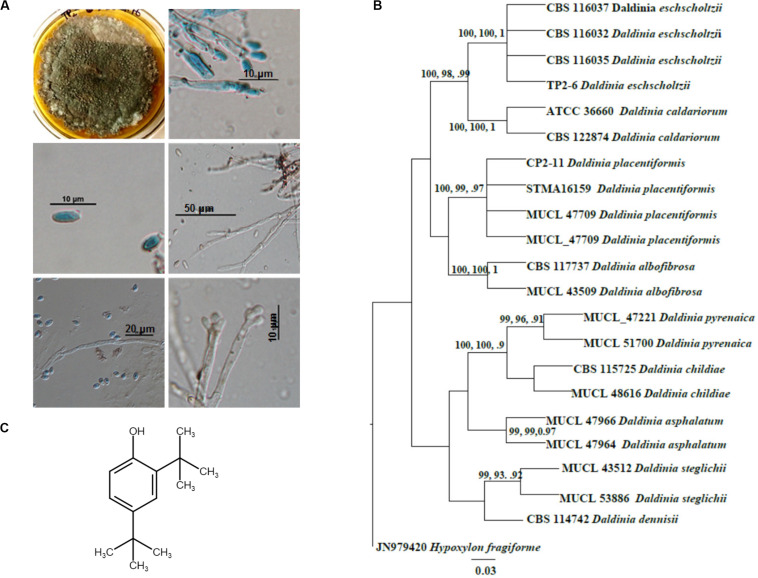
Identification and molecular phylogenetic analysis of *Daldinia eschscholtzii*. **(A)** Microscopic observations for the identification of *D. eschscholtzii*. The colony morphology, mycelia, and spores stained in lactophenol cotton blue are shown. **(B)** Phylogram based on the RAxML analysis of the ITS-1 DNA sequence dataset. Bootstrap support values for ML, MP higher than 75%, and BYPP values greater than 0.90 are given above each branch respectively for *D. eschscholtzii* (TP2-6, an in-house code given to the isolate). **(C)** Molecular structure of 2,4-di-tert-butylphenol (2,4-DBP) identified as the bioactive compound that was isolated from *D. eschscholtzii.*

A mature colony of TP2-6 was olivaceous green with smoky gray appearance at the surface texture. The margin was as follows: entire; colony fluffy in texture; surface color changes from white to dark gray and covers entire plate (90 cm) after 6 days of inoculation. The culture produced spores, which imparts a grainy appearance to the surface. Light microscopy revealed the hyphae to be septate, hyaline, to melanized thick walled as the colony ages. The conidia produced were small, numerous, hyaline, and ellipsoid with an attenuated base (Figure 1A). Apart from the morphological observations, the sequencing data confirmed it as *Daldinia eschscholtzii*. The sequence obtained in molecular analyses was submitted to GenBank with accession number KX987249.

Phylogenetic analysis of the sequence data consisted of Bayesian and maximum likelihood analysis as combined aligned dataset. The ITS dataset comprised 22 taxa and 647 characters from *Daldinia* species with *Hypoxylon fragiforme* as an outgroup. RAxML analysis of the ITS dataset yielded a best-scoring tree with a final maximum likelihood of 2060.50. In the maximum parsimonious dataset, of 647 total characters, 58 variable characters are parsimony-uninformative, and the number of parsimony-informative characters is 112. The parsimony analysis resulted in 10 equally parsimonious trees with a length of 253 steps (CI = 0.798, RI = 0.891, RC = 0.712, HI = 0.202). Bootstrap values of ML and MP equal to or above 75% based on 1000 replicates were shown (Figure 1B). Trees generated under maximum likelihood (ML), maximum parsimony (MP), and Bayesian analyses were similar in topology. The phylogenetic analyses show that our taxon groups together with *D. eschscholtzii* share a sister relation with *D. placentiformis*, *Daldinia caldariorum*, and *Daldinia albofibrosa* (99% ML/100 MP/1BYPP).

### 2,4-Di-Tert-Butylphenol Was Identified as the Bioactive Anti-QS Compound

Further, we were interested in isolating the bioactive compound from a crude extract of *D. eschscholtzii.* The crude extract from the *D. eschscholtzii* culture was prepared and subjected to column chromatography with increasing polarity from hexane to water with different ratios of solvents. Nine fractions were collected separately, which were subjected to bioactivity-guided fractionation. Active fractions were subjected to column chromatography with hexane: ethyl acetate (7:3) ratio, and fractions were collected in 20-mL volume aliquots. A single band was observed on the TLC plate from a purified fraction, and the purity was confirmed from analytical HPLC using the C_18_ column, using photodiode array detectors (PDA) with model number SPD-M20A (Shimadzu, Japan) (Supplementary Figure S1). HPLC analysis of the purified sample revealed a major component of ≥95% and a few minor impurities. The purified fraction was subjected to high-resolution mass spectrometry (HRMS) to estimate the molecular weight of the compound (Supplementary Figure S2A). The molecular weight was established by Q-TOF (quadruple-time-of-flight) HRMS mass spectrometry, with mass of [M + H] + at m/z 207.17, corresponding to the major peak, which gives the accurate mass of ≈206.17, in the positive ion mode (Supplementary Figure S2A). FTIR spectra of a purified sample displayed a peak at 3511 cm^–1^, which indicates stretching of the O–H phenolic group. Further, C–C stretch of the alkyl group was represented by the peaks at 2863–2951 cm^–1^. Moreover, the peak observed at 1247 cm^–1^ reveals a C–O stretching of phenols. An aromatic C–C stretch was recognized by peaks at 1504–1604 cm^–1^ (Supplementary Figure S2B). Thus, evidences provided by the above functional groups confirmed the phenolic nature of the compound.

^1^H NMR data of the purified sample shows the occurrence of two singlets at 1.325 ppm and 1.447 ppm which resembles a di-substituted tertiary butyl group (Supplementary Figure S3). Another singlet around 4.662 indicates the presence of a phenolic hydrogen. The rest of the 3 protons were detected in the aromatic downfield region in between 6.602 and 7.332 ppm, which suggests it to be a tri-substituted aromatic compound. This data was further supported by ^13^C NMR. The ^13^C NMR spectra of the isolated pure compound exhibited the occurrence of 10 carbon signals, of which 6 were downfield carbon signals present in between 116.07 and 151.89 ppm which were in the aromatic region. The remaining four carbon signals were detected in the upfield region in between 29.81 and 34.87 ppm. Out of six carbons, the aromatic substitution was confirmed by the presence of three quaternary carbons at 135.32 ppm, 143.12 ppm, and 151.89 ppm. Moreover, the presence of a phenolic OH group at quaternary carbon present at the most downfield region, i.e., 151.89 ppm, was confirmed in the molecule. Among the four carbon signals present in upfield regions, two were tertiary carbons at 34.87 ppm and 34.42 ppm while the rest of the two were methyl signals at 331.77 ppm and 29.81 ppm. Thus, the spectrum shows that in the compound, two tert-butyl substitutions are present on the remaining quaternary carbons at 135.32 ppm and 143.12 ppm in the aromatic ring. Therefore, after the interpretation of the hydrogen and carbon spectra, the structure of the compound was structurally elucidated as 2,4-di-tertbutylphenol (C_14_H_22_O) (Figure 1C). The final yield of 2,4-DBP (≥95% purity) was 1.9 mg/L.

### 2,4-DBP Shows Anti-QS Activity Against *C. violaceum* and *P. aeruginosa*

**FIGURE 2:**
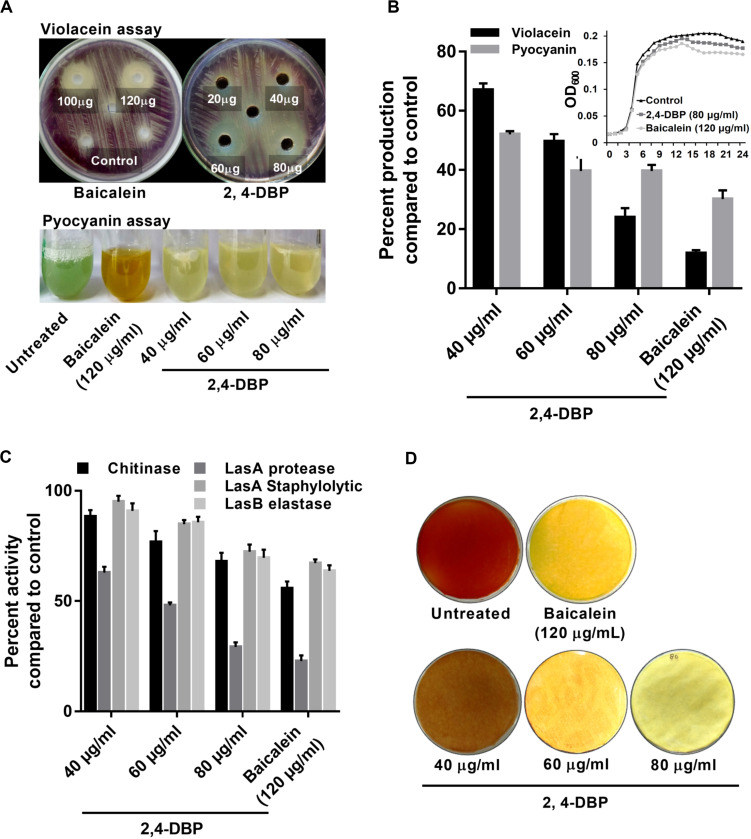
Inhibition of virulence factors under control of quorum sensing by 2,4-di-tertbutylphenol (2,4-DBP). **(A)** Visual qualitative assay for inhibition of *Chromobacterium violaceum* violacein production and *Pseudomonas aeruginosa* pyocyanin production by 2,4-DBP at indicated concentrations. **(B)** Quantitative estimation of violacein and pyocyanin production. The growth curve in the presence of 2,4-DBP, baicalein, and untreated control is shown in the inlet. **(C)** Inhibition of chitinase, LasA protease, LasA staphylolytic activity, and LasB elastase activities by 2,4-DBP at indicated concentrations. **(D)** Visual qualitative assay of inhibition of *P. aeruginosa* HCN production by 2,4-DBP at indicated concentrations. Bar diagrams represent mean percentage values of triplicates normalized with those obtained with untreated controls.

The expression for the production of the violacein pigment by *C. violaceum* is regulated by QS. Therefore, any inhibitor of *C. violaceum* can be visually determined by the inhibition of production of the pigment. Hence, it has been used as a marker trait in QS studies as a reporter model (Stauff and Bassler, 2011). In our study, 2,4-DBP and BAC (positive control) were able to inhibit violacein production in the reporter strain on the agar plate (Figure 2A). The zone of inhibition of violacein production was 16 mm diameter for 2,4-DBP (80 μg/mL) and 18 mm for BAC (120 μg/mL). When expressed quantitatively, inhibition of violacein production when treated with 2,4-DBP (76% at 80 μg/mL) was comparable to that when treated with BAC (88% at 120 μg/mL) Figure 2B.

In earlier studies, inhibition zones of 10 mm and 13 mm of violacein production were reported for two phenethylamide metabolites isolated from marine *Halobacillus salinus* bacteria (Teasdale et al., 2009). In a more recent study, Noumi et al. (2018) reported 69.3% of violacein inhibition by tea tree oil. Our study achieved a stronger inhibition of violacein production by 2,4-DBP that we isolated from *D. eschscholtzii*. Since the molecule showed anti-QS activities, we hypothesized that this compound can inhibit growth of the notorious human nosocomial pathogen *P. aeruginosa*, or its virulence factors that are controlled by QS.

### 2,4-DBP Does Not Inhibit Growth of *P. aeruginosa*

The MIC based on the microbroth dilution method was calculated for both 2,4-DBP and BAC to examine if 2,4-DBP shows any inhibitory effect on the growth of *P. aeruginosa*. The MIC for 2,4-DBP was found to be >1024 μg/mL (not shown here). The growth pattern and change in the cell density of *P. aeruginosa* also remained largely unaffected when treated with three different concentrations: 40, 60, and 80 μg/mL of 2,4-DBP. This suggests that 2,4-DBP has no effect toward the growth kinetics of the bacterium, when compared to the growth in the presence of the sub-MIC level of BAC (120 μg/mL) (Figure 2B). This result was well in agreement with a similar observation that was reported by Viszwapriya et al. (2016) where 2,4-DBP showed a non-bactericidal effect on the growth of *Streptococcus pyogenes*.

Though the compound showed no effect toward the growth of the pathogen, inhibition of virulence factors of *P. aeruginosa* that are controlled by QS could be an invaluable potential of 2,4-DBP, especially when it showed strong anti-QS activities. Therefore, we intended to study the effect of 2,4-DBP on the expression of QS-regulated genes and QS-regulated production of extracellular virulence factors, production of biofilm and its associated factors, and *in vitro* host-cell adhesion and invasion. The same concentrations of 2,4-DBP (40, 60, and 80 μg/mL) and BAC (120 μg/mL) were used throughout the rest of the study.

### 2,4-DBP Treatment Greatly Reduced Pyocyanin Production in *P. aeruginosa*

Pyocyanin, a predominant green-colored phenazine pigment and a redox-active toxin secreted by *P. aeruginosa*, critically plays a detrimental role for the establishment of an infection. The Rhl component of the QS system in *P. aeruginosa* activates the expression of pyocyanin production in conjunction with RhlR and the autoinducer signal molecule C4-HSL (Bratu et al., 2006). Pyocyanin also induces pathogen-driven neutrophil apoptosis by reducing local inflammation and creates a biofilm formation environment (Allen et al., 2005). In our study, 2,4-DBP treatment reduced the level of pyocyanin production by 60% whereas BAC reduced it by 69% without significantly affecting the bacterial growth (Figure 2B). This can also be visualized by the abrupt decrease in green color pigment in the supernatant of treated cultures as compared to those untreated (Figure 2A). This reduction was superior to those achieved by one previous study, wherein ethanolic extract, ethyl acetate extract, and N-butanol extract of *Camellia nitidissima* Chi flower at a concentration of 0.75 mg/mL reduced pyocyanin production by 51.2%, 56.9%, and 51.5%, respectively (Yang et al., 2018).

### 2,4-DBP Treatment Considerably Reduced Chitinase Activity *P. aeruginosa*

Chitinolytic activity by bacteria plays a significant role in chitin degradation, which results in recycling of a carbon as well as nitrogen source into a simply accessible form in the ecosystem (Gooday, 1990). The expression of chitinase enzyme enhances in clinical isolates, thus playing a role in virulent infection (Salunkhe et al., 2005). In our study, we recorded 2,4-DBP and BAC at concentrations of 80 and 120 μg/mL, respectively, to reduce chitinase activity in the culture extracts of *P. aeruginosa* (Figure 2C). The reduction in chitinase activity was found to be 27.1% and 30.8% in the case of 2,4-DBP and BAC, respectively, as compared to the untreated control. Interestingly, however, Husain et al. (2013) reported 80% inhibition in chitin production when *P. aeruginosa* was treated with 1.6% of clove oil.

### 2,4-DBP Causes Dose-Dependent Decrease in *P. aeruginosa* Protease Activity

Initial establishment of infection in host tissues is instigated by elastases and proteases. *P. aeruginosa* secretes several protease and elastase virulence factors regulated by LasIR, which implies their roles in its pathogenicity (Kessler et al., 1993). Elastase, a powerful T2SS-secreted proteolytic enzyme, is encoded by gene lasB. It has a wide range of substrates, including elements of connective tissue such as elastin, collagen, fibronectin, and laminen. These bacterial proteases act as hydrolytic enzymes that target the host’s proteins to facilitate the invasion and growth of the pathogen (Musthafa et al., 2011). In *P. aeruginosa*, expression of exoproteins such as alkaline protease and elastase is under the regulation of QS (Swift et al., 1996). In light of the ability of 2,4-DBP as a QS inhibitor, we investigated the role of 2,4-DBP on azocasein-degrading protease activity, LasA stapylolytic activity, and LasB elastase activity and observed a dose-dependent decrease in the protease activity of *P. aeruginosa* (Figure 2C). 2,4-DBP reduced LasA staphylolytic activity by 27.6% at 80 μg/mL and BAC by 32.8%. Equivalently, LasB elastase activity was attenuated by 30% as compared to BAC by 36.3%. Furthermore, LasA protease activity was found to get attenuated by 70.7% due to 2,4-DBP at 80 μg/mL whereas BAC reduced this to 77.1%. Although Rajkumari et al. (2018b) reported the decrease in staphylolytic activity by 21.8% and LasA protease by 71% when treated with cinnamic acid, in the present study, 2,4-DBP exerted moderate effects on LasA staphylolytic activity and LasB elastase attenuation but significant reduction in LasA protease activity.

### 2,4-DBP Inhibits *P. aeruginosa* HCN Production

Production of HCN provides an advantage to *P. aeruginosa* to inhabit a range of ecological niches and hence contribute to its pathogenicity (Williams et al., 2006). Cyanide promptly diffuses in tissue and inhibits aerobic chain reaction by irreversibly binding to the terminal oxidases of respiratory chains and hence its profound toxicity (Zlosnik et al., 2006). In our study, treatment with 2,4-DBP resulted in attenuation of HCN production in contrast to untreated control. The HCN thus produced reacts with picric acid (yellow in color) in the presence of sodium carbonate, resulting in a color change from yellow to orange to brick red. A sharp decrease in color change of filter paper from yellow to orange depicted less HCN production in case of 2,4-DBP and BAC at 80 and 120 μg/mL, respectively, whereas a deep-orange color was observed in drug-free control depicting high HCN production (Figure 2D).

### 2,4-DBP Strongly Inhibits Motility of *P. aeruginosa*

**FIGURE 3:**
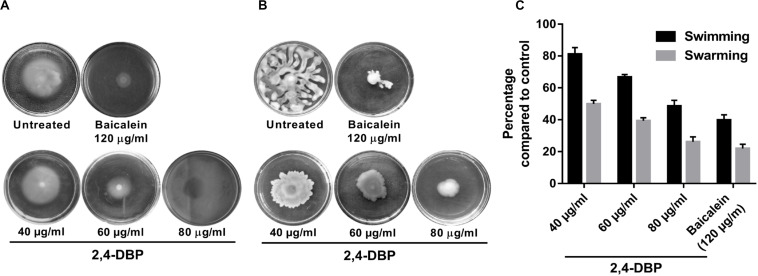
Inhibition of *Pseudomonas aeruginosa* swimming motility **(A)** and swarming motility **(B)** by 2,4-di-tert-butylphenol (2,4-DBP) at the indicated concentrations. **(C)** Quantitative estimations of the inhibition of *P. aeruginosa* swimming and swarming motility by 2,4-DBP. Bar values indicate mean percentages of triplicates normalized with those obtained with untreated controls.

*Pseudomonas aeruginosa* possesses an exquisite mechanism to ingeniously use different types of motilities to facilitate colonization in several ecological niches. Motility of *P. aeruginosa* also plays a significant role in the surface attachment and maturation of biofilms (O’toole and Kolter, 1998). *P. aeruginosa* possesses a polar flagellum that aids in swimming and swarming on liquid and semisolid surfaces, respectively (Murphy, 2009; Murray et al., 2010). Herein, we investigated the swimming and swarming ability of *P. aeruginosa* when treated with 2,4-DBP and BAC. As evident from Figures 3B,C, the inhibition of swarming was 78% when treated with 2,4-DBP at 80 μg/mL, while the positive control BAC inhibited swarming by 73.9% at 120 μg/mL. Similarly, when treated with 2,4-DBP at 80 μg/mL, swimming was inhibited by 60.2%, while BAC reduced this to 51.5% (Figures 3A,C), suggesting that 2,4-DBP shows higher inhibitory effects on motility of *P. aeruginosa* than BAC does. According to earlier studies, the inhibitory effect of aspirin on the swimming motility of *P. aeruginosa* was 34% (El-Mowafy et al., 2014). Similarly, Li et al. in 2018 reported that cinnamaldehyde could restrict swarming up to 58.4% and swimming up to 40.7% at the concentration of 1 μL/mL. Compared to these earlier results, we could achieve greater inhibition of swimming as well as swarming motility of *P. aeruginosa* by 2,4-DBP.

### 2,4-DBP Attenuates *P. aeruginosa* eDNA Production

**FIGURE 4:**
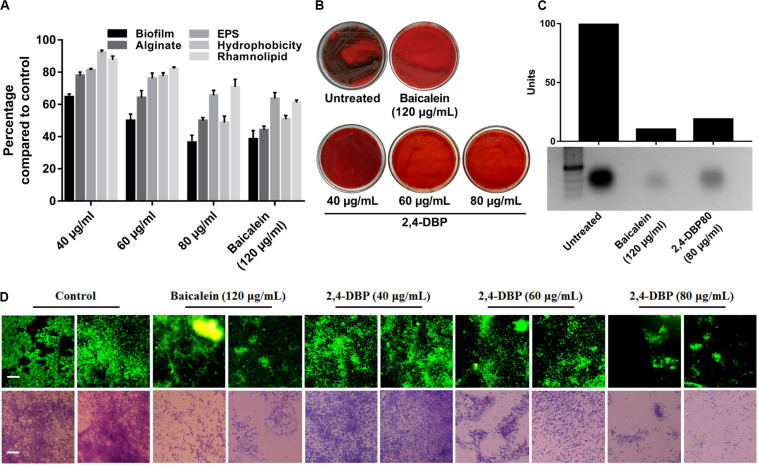
Inhibition of *P. aeruginosa* biofilm formation and biofilm-associated factors by 2,4-di-tertbutylphenol (2,4-DBP) at the indicated concentrations. **(A)** Inhibition of *P. aeruginosa* biofilm formation, production of alginate, exo-polysaccharide (EPS), and rhamnolipid, and hydrophobicity. Bar values indicate mean percentages of triplicates normalized with those obtained with untreated controls. **(B)** Congo red agar visual qualitative assay for inhibition of biofilm formation. **(C)** Inhibition of eDNA production by baicalein and 2,4-DBP. The bar values show intensity of the bands as estimated by ImageJ software, from the agarose gel electrophoresis image shown below. **(D)** Fluorescence and light microscopic images of biofilms when treated with various concentrations of baicalein and 2,4-DBP. Two random representative images are shown for each treatment. The scale bars are equivalent to 25 μm.

Extracellular DNA (eDNA) is a major constituent of the biofilm matrix of *P. aeruginosa*. eDNA is supposed to be produced from random chromosomal DNA from dead bacteria gibe strength to the biofilm matrix (Allesen-Holm et al., 2006). During starvation, eDNA acts as a nutrient source of *P. aeruginosa* (Finkel and Kolter, 2001). Extracellular DNA, furthermore, is known to ease the biofilm expansion mediated by twitching motility as it maintains organized cell arrangements to synchronize the movement of cells (Gloag et al., 2013). In this study, we observed a significant decrease in *P. aeruginosa* eDNA production when treated with 2,4-DBP (Figure 4C). The reduction of eDNA was recorded to be ∼82%, which is on par with the BAC, as quantified with ImageJ software.

### 2,4-DBP Significantly Impairs *P. aeruginosa* Biofilm Formation

Biofilms are contemplated as a 3D network of microbial communities adhering to biotic or abiotic surfaces, enveloped by an extracellular matrix comprised of bacterium-derived DNA, exopolysaccharides, and proteins released by the bacteria embedded therein (Chen et al., 2018). Biofilms are clinically one of the most relevant features expressed by bacteria, since they act as an impermeable barrier to antibiotics and the host immune system, thus promoting antibiotic tolerance and persistence. The best known activator signal for biofilm formation is the QS signaling system. Hence, we investigated and quantified the deleterious effect of 2,4-DBP on biofilm formation by this bacterium. Notably, when compared to drug-free control, 2,4-DBP 80 μg/mL attenuated biofilm formation by 49%, marginally less (53%) than when treated with the positive control BAC at 120 μg/mL (Figure 4A). In the treatment, in contrast to 53% attenuation by BAC, biofilm attenuation by 2,4-DBP at a sub-MIC of 80 μg/mL was 49%, little less than the positive control. Disruption of the biofilm could also be directly observed under light microscopy when treated with 2,4-DBP. This can also be visualized on Congo red agar (CRA), which serves as a qualitative detection method for biofilm-positive bacteria. Bacteria that produce biofilms grow into dry crystalline black colonies when inoculated on a Congo red agar medium; they remain pink otherwise (Freeman et al., 1989). We observed the absence of black colonies of *P. aeruginosa* on Congo red agar when treated with 2,4-DBP, which is suggestive of the inability of the bacterium to produce a robust biofilm in the presence of test compounds (Figure 4B). A similar observation of biofilm with the help of light as well as fluorescent microscopy in Figure 4D corroborates with the fact that treatment with 2,4-DBP impairs the growth of biofilm development in the initial phase itself and inhibits biofilm development as compared to control.

In earlier reports, reduction in biofilm by phenolic compounds such as cinnamic acid, ferulic acid, and vanillic acid was 44, 45, and 46%, respectively (Ugurlu et al., 2016), suggesting 2,4-DBP as a potential anti-biofilm candidate. Similarly, Padmavathi et al. (2015) evaluated anti-fungal and anti-biofilm efficacy of 2,4-DBP and observed a strong anti-fungal action by inhibiting and disrupting biofilm formation in *Candida albicans*. Our results are also supported by reports of 2,4-DBP displaying a concentration-dependent biofilm inhibition that can reach a maximum of 79% 48 μg/mL concentration (Viszwapriya et al., 2016) in *Streptococcus pyogenes*. In addition, in a more recent study, Rajkumari et al. (2018b) described that the treatment with betulin and betulinic acid possessing an anti-QS ability resulted in the formation of pink colonies of *P. aeruginosa* on Congo red agar and hence reduced biofilm formation.

#### 2,4-DBP Significantly Inhibits Production of Exo-Polysaccharides

Extracellular polymeric substances (EPS) are the main constituent of *P. aeruginosa* biofilms and crucial for its biofilm architecture (Kreft and Wimpenny, 2001). EPS helps *P. aeruginosa* to evade antibiotic treatment and immune responses (Ghafoor et al., 2011). Besides providing mechanical stability to biofilm through various interactions, EPS defend bacterial cells by impeding penetration and/or sequestering of antimicrobial agents (Donlan, 2002; Ryder et al., 2007). In our study, we found that, when bacterial cells were exposed to 2,4-DBP, the production of EPS was reduced by 34.4%, while the reduction was 36% for BAC (Figure 4A). This marked decrease on par with the positive control reflects the ability of 2,4-DBP as a potential anti-biofilm candidate. Similar reports were also present wherein the decrease in EPS was 31.2% by botulin, 18% by betulinic acid, and 31% by clove oil (Husain et al., 2013; Rajkumari et al., 2018b). In addition, 2,4-DBP inhibited EPS production by *Candida albicans* by 33%, (Padmavathi et al., 2015) and up to 33–46% in *Streptococcus* sp. (Viszwapriya et al., 2016), which are similar to that of our results.

#### 2,4-DBP Causes Significant Reduction of *P. aeruginosa* Rhamnolipid Production

Rhamnolipid is an extracellular virulent factor and a prerequisite for biofilm establishment. It actively maintains *P. aeruginosa* biofilm architecture and reduces adhesion between bacterial cells (do Valle Gomes and Nitschke, 2012). In their involvement in early cell-to-surface interactions, further maintenance following dispersion/disruption of the biofilm is indispensable (Davey et al., 2003). Rhamnolipid is a biosurfactant composed of a rhamnose-containing glycolipid detergent-like structure and is well known to solubilize the phospholipids of lung surfactant, hence more prone to cleavage by phospholipase C (Köhler et al., 2010). We witnessed a reduction in rhamnolipid production when exposed to 2,4-DBP by 29.2%, compared to 38.8% for BAC (Figure 4A). Our study achieved better reduction in rhamnolipid production when compared to previous reports, wherein 19.03% and 21.61% reductions in rhamnolipid production were achieved when treated with betulin and betulinic acid at 125 μg/mL concentration (Rajkumari et al., 2018b).

#### 2,4-DBP Impedes *P. aeruginosa* Alginate Secretion

*Pseudomonas aeruginosa* secretes alginate, a major polysaccharide component of the *P. aeruginosa* EPS component, which determines its surface characteristics such as hydrophobicity, charge, and electrostatic interactions of the cell surface with the surface (Herzberg et al., 2009). Alginates shield bacteria from adverse conditions and enhance surface adhesion (Boyd and Chakrabarty, 1995). Its production by *P. aeruginosa* aids in antibiotic resistance, phagocytic evasion, resistance toward macrophages and neutrophils, and scavenging of reactive oxygen intermediates (Cody et al., 2009). In our study, 2,4-DBP at 80 μg/mL impeded *P. aeruginosa* alginate secretion by 50%, while BAC treatment at 120 μg/mL resulted in a reduction by 55% (Figure 4A). This finding is significant as it corroborates with the previous reports of cinnamon oil, reducing alginate production by 54% at 0.2 μl/mL (Kalia et al., 2015).

#### 2,4-DBP Significantly Decreases *P. aeruginosa* Cell-Surface Hydrophobicity

Hydrophobicity on bacterial surfaces plays a determinant role in the adhesion and biofilm formation of bacterial pathogens on animate as well as inanimate surfaces (Rosenberg and Doyle, 1990). The ability of *P. aeruginosa* to adhere to hydrocarbons is a measure of cell-surface hydrophobicity. A greater CSH is suggestive of a greater ability of the bacterium to adhere. This is achieved by shielding the repelling forces amid the surface charges, which is critically needed for early micro-colony formation during biofilm development (Pamp and Tolker-Nielsen, 2007). Hence, CSH is regarded as a major determinant of biofilm formation (Silva-Dias et al., 2015). In this study, CSH was reduced by 51.2% and 49.2% when treated with 2,4-DBP and BAC, respectively, which suggest its role in inhibiting adhesion of *P. aeruginosa* (Figure 4A), which is suggestive of a reduction in biofilm formation of the pathogen, as noted in the above results. In a similar study, 2,4-DBP resulted in significant reduction up to 70% in cell-surface hydrophobicity of *Streptococcus* sp.

Since 2,4-DBP inhibited cell-surface hydrophobicity of *P. aeruginosa* considerably, we hypothesized that the test compound could also inhibit adhesion of the pathogen to its host cells.

### 2,4-DBP Downregulates QS Genes of *P. aeruginosa*

**FIGURE 5:**
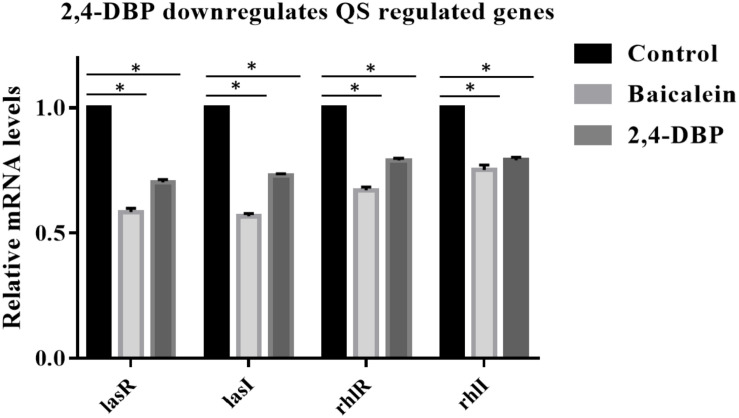
Relative expression of *P. aeruginosa* PAO1 *lasA*, *lasI*, *rhlR*, and *rhlI* genes under control conditions (DMSO) and following addition of 2,4-di-tert-butylphenol (2,4-DBP) (80 μg/mL). *indicates that *p* < 0.05 and hence statistically significant which was assessed by two tailed *t*-test.

Since 2,4-DBP strongly inhibits the QS and secretion of virulence factors of *P. aeruginosa*, we hypothesized that 2,4-DBP might inhibit the expression of QS-related genes. To investigate this, we quantified the expression levels of QS-associated genes such as *lasI*, *lasR*, *rhlR*, and *rhlI* in *P. aeruginosa* which are treated with 2,4-DBP by using quantitative RT-PCR. We found that the treatment of *P. aeruginosa* with 2,4-DBP at 80 μg/ml concentration decreased the mRNA level of all the four QS-associated genes *lasI*, *lasR*, *rhlR*, and *rhlI RhlR* significantly on par with the positive control BAC, a well-studied inhibitor of QS (Figure 5). These results suggest that 2,4-DBP inhibits QS by downregulating the expression of QS-related genes.

#### 2,4-DBP Causes Strong Inhibition of *P. aeruginosa* Host-Cell Adhesion

**FIGURE 6:**
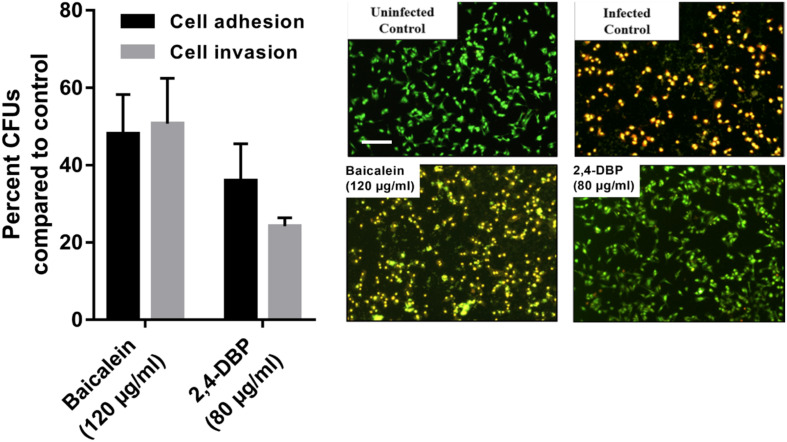
Inhibition of *P. aeruginosa* host-cell adhesion and host-cell invasion by 2,4-di-tert-butylphenol (2,4-DBP) in untreated control, baicalein, and 2,4-DBP in the A549 lung epithelial cell infection model (left). Live/dead imaging of A549 lung epithelial cell by AO/EB, when infected with *P. aeruginosa* PAO1 in untreated control, when treated with baicalein (120 μg/mL) and 2,4-DBP (80 μg/mL) (right). The scale bar is equivalent to 20 μm.

Host-cell adhesion of *P. aeruginosa* is the initial and decisive stage of colonization in the host and is crucial for an infection by the bacterium to be established. To evaluate whether 2,4-DBP impairs host-cell adhesion of *P. aeruginosa*, we infected A549 human alveolar carcinoma cells with *P. aeruginosa* in the presence of 2,4-DBP and BAC. The adhered cells were harvested and enumerated by CFU counts. We recorded an abrupt reduction in *P. aeruginosa* host-cell adhesion by 72% in the presence of 2,4-DBP at 80 μg/mL, as compared to 62% reduction in the presence of the positive control BAC at 120 μg/mL (Figure 6). The reduction in host-cell adhesion achieved in our study was remarkably higher than those achieved in previous studies, wherein it was 26.3% by the antibiotic ciprofloxacin at a concentration of 0.063 μg/mL, 16.4% by dextran at 5 mg/mL, 45.2% by an extract of soybean at 4.3 mg/mL, and 54.5% by a cranberry extract at 2.6 mg/mL (Ahmed et al., 2014). This makes 2,4-DBP an attractive candidate for anti-virulence therapeutic strategies, whereby the pathogen can be sensitized to antimicrobials and/or the host’s immune system, especially when biofilm-related infection is widespread and multidrug resistance in *P. aeruginosa* is rampant.

#### 2,4-DBP Causes Severe Impairment of *P. aeruginosa* Host-Cell Invasion

*Pseudomonas aeruginosa* is known to escape the host’s immune system by promoting its own internalization into host non-phagocytic host cells (Chi et al., 1991; Engel and Eran, 2011). To evaluate if 2,4-DBP is able to impair *P. aeruginosa* host-cell invasion, we infected A549 cells with *P. aeruginosa in vitro* at a multiplicity of infection of 100 in the presence of 2,4-DBP and BAC. The non-internalized bacteria were killed by gentamicin treatment; the internalized bacterial cells were harvested and enumerated by CFU counts. In the presence of 2,4-DBP at 80 μg/mL, host-cell invasion was severely reduced by 75%, whereas this reduction was 50% when infected in the presence of the positive control BAC at 120 μg/mL (Figure 6). Such a remarkable reduction in the host-cell invasion of bacterial cells depicts the potential of 2,4-DBP when compared to other studies, wherein invasion was decreased by about 45% when treated with ciprofloxacin at the concentration of 0.063 μg/mL, and 25% in the case of dextran at 5.0 mg/mL. However, a significant reduction was achieved when treated with an extract of soybean at 4.3 mg/mL, in combination with ciprofloxacin and dextran (Ahmed et al., 2014).

#### 2,4-DBP Interferes With Host-Cell Death by *P. aeruginosa*

The ability of *P. aeruginosa* getting internalized eventually leads to induction of apoptosis, which is the tangible virulence of the bacterium resulting in host tissue damage. To investigate if our test compound can protect the host cells from the induction of apoptosis induced by the internalized bacteria, we infected the host cells with the pathogen at a multiplicity of infection of 100, followed by elimination of the extracellular bacteria. Host-cell death was observed by live dead cell imaging after 24 h of incubation. The cells were stained briefly with Acridine Orange/Ethidium Bromide solution and directly observed by fluorescence microscopy. Our results depict that bacterial cells treated with BAC resulted in more A549 cell deaths after 24 h of incubation while the degree of death induced in 2,4-DBP at the 80-μg/mL treatment was less (Figure 6).

### Synergistic Studies of 2,4-DBP With Antibiotics

**FIGURE 7:**
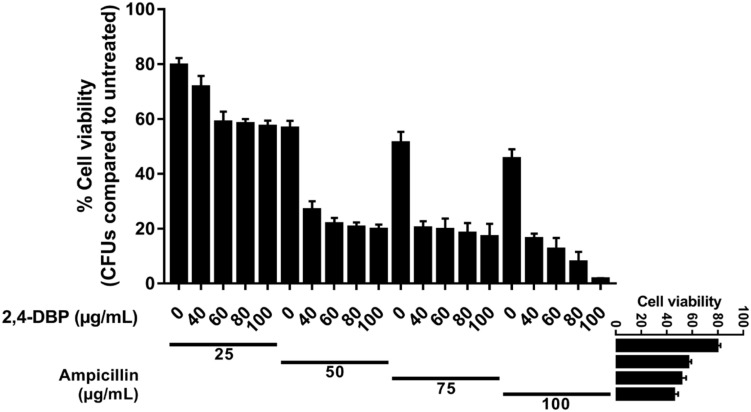
Synergistic studies of 2,4-di-tert-butylphenol (2,4-DBP) with ampicillin: Graph showing cell viability of *P. aeruginosa* when treated with ampicillin and combination of ampicillin and 2,4-DBP.

The MIC of ampicillin and 2,4-DBP was found to be more than 1024 μg. It means *P. aeruginosa* was resistant to the antibiotic and grows without any constraints even in the presence of 2,4-DBP at 80 μg/mL, as mentioned earlier. We were interested to investigate the combined effect of ampicillin and 2,4-DBP on *P. aeruginosa* and hence several combinations of ampicillin and 2,4-DBP were used in different concentration. As shown in Figure 7, the combination of ampicillin at 100 μg/mL and 2,4-DBP at 100 μg/mL was effective in eradicating the bacterial growth as the bacterial cell viability at this combination was less than 2%. Furthermore, as evident from Figure 7, even the effect of concentration of ampicillin at 50, 75, and 100 μg/mL alone is similar and ineffective in killing *P. aeruginosa*. However, the introduction of 2,4-DBP even at the 40-μg/mL concentration in combination with ampicillin results in sharp deleterious effects on the bacterium. Hence, it could be presumed that 2,4-DBP was capable of weakening the bacterial cells, and further, ampicillin was able to kill the weakened pathogen, which earlier was ineffective at even higher concentrations. A similar sort of study was performed by Viszwapriya et al. (2016), where 2,4-DBP reduced the MIC of the standard antibiotic. A marked decrease in MIC value of erythromycin and tetracycline was observed in combination with 2,4-DBP against *Streptococcus* sp.

#### Docking Analysis of 2,4-DBP

The three-dimensional structure of LasR was retrieved from the PDB database. Bottomley et al. (2007) reported the crystal structure of the LasR receptor protein at 1.80 Å resolution. The NCBI CD database search of this protein revealed that it contains an autoinducer domain from residues 20 to 160, which is crucial for the transcription process (Marchler-Bauer et al., 2010). The complete structure details of this protein is discussed by Gopu et al. (2015). As the three-dimensional structure of RhlR is not solved, the predicted model, which was reported earlier (Rajkumari et al., 2018b), was used in this study. Molecular docking studies were performed to find out the hotspot residues of the protein.

**FIGURE 8:**
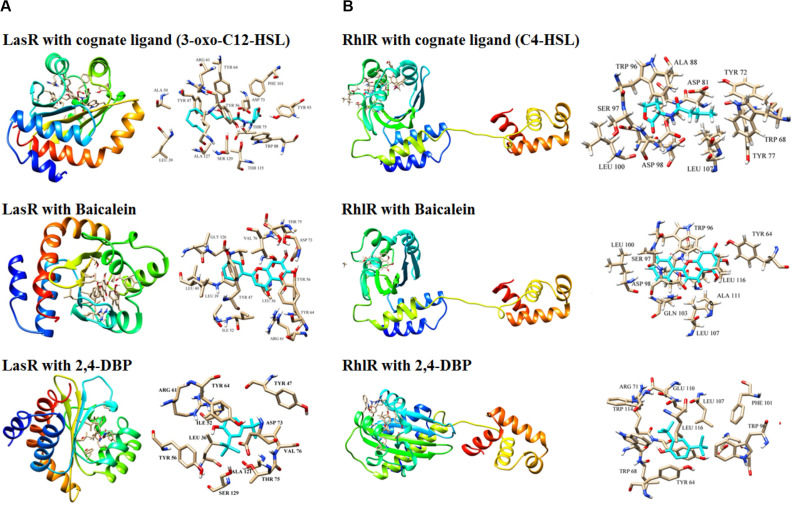
Docking of LasR **(A)** and RhlR **(B)** with their cognate ligands (3-oxo-C12-HSL and C4-HSL, respectively), baicalein and 2,4-di-tertbutylphenol (2,4-DBP). Three-dimensional structures of the LasR–cognate ligand complex and RhlR–cognate ligand complex are shown at the top of both the panels. For each complex, stick representations of the ligand molecule, along with its interacting protein residues, are shown on the right of each panel.

Ligand molecule, 2,4-DBP, was docked in order to study the inhibition mechanisms. The signaling molecule showed less dock scores as compared to the ligand molecules (2,4-DBP and BAC). Information on all the interacting atoms of protein and ligands along with H-bond distances is provided in Supplementary Table S4. The pose of ligands in the complex with LasR and RhlR receptor proteins is shown in Figures 8A,B, respectively. This docking study revealed the hotspot residues of protein, which interacted with ligands. Few other residues were also noticed to interact with ligands and were highlighted in bold.

#### Molecular Dynamics Simulation

**FIGURE 9:**
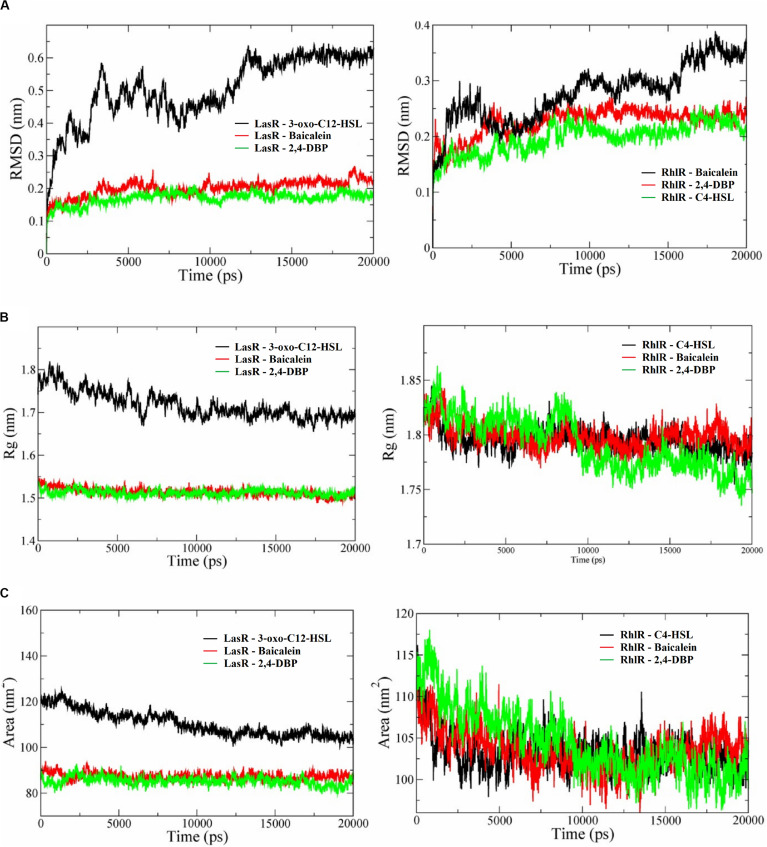
**(A)** Root mean square deviation (RMSD) profiles **(A)**, radius of gyration graphs **(B)**, and SASA (solvent-accessible area) graphs **(C)** of *P. aeruginosa* LasR and RhlR receptor proteins complexed with their cognate ligands 3-oxo-C12-HSL and C4-HSL, respectively, baicalein and 2,4-di-tertbutylphenol (2,4-DBP). In all the graphs, the receptor protein is less stable than with baicalein and 2,4-DBP.

Molecular dynamics simulation studies were performed to study the conformational changes in proteins’ three-dimensional structure for activation and deactivation of the LasR receptor protein in the presence of respective ligands. The simulations were performed with six complexes, LasR + signaling, LasR + BAC (LasR + BAC), LasR + 2,4-DBP, RhlR + 2,4-DBP, RhlR + BAC, and RhlR + 2,4-DBP. RMSD profiles of all simulated complexes were generated to study the protein deviation throughout the simulation period. The simulations were run for 20 ns with the time step of 10 ps and are shown in Figure 9A. The RMSD profile of this protein revealed that the protein-signaling molecule complex showed more deviation as compared to other two complexes. The same pattern of deviation was also revealed by three complexes of the RhlR protein. This instability in the three-dimensional conformation was caused because the crystal structure of the signaling molecule was crystallized with the signal molecule, which handles the activation of the LasR protein. The protein + BAC and ligand complexes showed the least deviation in comparison to the protein-signaling molecule.

Deviation in RMSD profile in a protein can occur for several possible reasons; it can be due to either expansion or contraction of the protein, or it may be because of folding of protein in the other direction. RMSD data was followed by analysis of radius of gyration (Figure 9B) to understand the deviation pattern of proteins. The expansion showed by the protein-signaling molecule was higher among all simulated complexes, whereas it was less in protein–ligand complexes. Both the proteins consist of the same cavity as an active site. The activation of the LasR protein depends on the availability of this cavity for the signaling molecule. SASA (solvent-accessible surface area) graphs (Figure 9C) were generated to study how accessibility of this protein is affected due to opening or closing of the binding site. Keen observation of the SASA graph revealed that the protein had lost its surface accessibility when it interacts with 2,4-DBP.

*In silico* analysis provides insights into protein three-dimensional structures at the atomic level. The structural details of protein and ligands provide more details to improve the efficiency of drugs. A molecular docking study of the LasR receptor protein with both 2,4-DBP and signaling molecules revealed that they bind rigidly to the receptor. The structural comparison of both signaling molecules revealed that they shared a similar backbone. The functional groups of both molecules are different, and hence, their interaction pattern is different. The interaction of this functional group with protein residues may lead to these conformational changes. The RMSD profile of all the complexes showed that the protein-signaling complex of both proteins was the most dynamic complex whereas the protein + 2,4-DBP complex is the most rigid complex found in both the cases. The least deviation shown by the protein + 2,4-DBP complex indicates a stable complex and hence suggests this as a potential inhibitor of both the proteins. A similar pattern shown by the 2,4-DBP molecule as well as known inhibitor molecules proves that this ligand may be a potential inhibitor. The radius of gyration and SASA study revealed that this 2,4-DBP interacts with proteins strongly. The interactions of ligand had induced confirmation changes in the LasR receptor in such a way that the active site is no longer available to interact with other molecules. It was proved through molecular docking and molecular dynamics simulation studies that 2,4-DBP can act as an anti-quorum agent against *P. aeruginosa*.

## Summary and Conclusion

To face the recurring breakdown of antibiotics success against *P. aeruginosa* infections, recent efforts have switched toward exploiting QS inhibitors as anti-pathogenic strategy. The prospective of this strategy is encouraging since the majority of such compounds are of natural origin produced by organisms. More efforts are now being put in this concept, and it is acquiring momentum, being apparent from the extensive studies correlated with anti-QS subjects. In the present study also, 2,4-DBP, a proposed anti-QS compound, was isolated from *D. eschscholtzii*, a foliar endophytic fungus associated with *T. procumbens*. *D. eschscholtzii* in general is a wood-inhabiting/decaying endophytic fungi prevailing in warm tropical climate. On account of their endophytic habit, *Daldinia* sp. possess the trait of early colonization (Stadler et al., 2014). This fungus manifests a multitude of secondary metabolites as evident from fascinating data in the last decade (Ng et al., 2016). To this end, the isolated 2,4-DBP was assessed for its anti-QS activity and anti-biofilm activity against *P. aeruginosa*, a well-known opportunistic pathogen. We found that 2,4-DBP showed significant activity in inhibiting the production of various virulent factors such as pyocyanin, chitinase, several proteases, and biofilm-associated factors along with deleterious effects on biofilm formation. The Las and Rhl systems are closely related and are known to control the development of various virulence factors including alkaline protease, elastase, exotoxin A, lectins, pyocyanin, rhamnolipids, superoxide dismutase, and biofilm formation (Venturi, 2006). There is no direct evidence for swimming motility and alginate synthesis under the control of QS (Ledgham et al., 2003). However, along with the other virulence factors, these two phenotypes are often affected when QS is inactivated or inhibited (Bala et al., 2011; Gupta et al., 2016), hence suggesting that the regulatory modules responsible for these two phenotypes may at least partially overlap with the QS signaling network (Luo et al., 2016).

*In silico* studies also showed its ability to bind to QS-regulated receptor proteins, Las and Rhl, and inhibit the binding of cognate signal molecules to inhibit QS. In earlier reports, 2,4-DBP is reported to curtail the killing of *Caenorhabditis elegans* up to 73%, when infected with *Streptococcus pyogenes*, while *S. pyogenes* was capable of killing 100% *C. elegans* after 96 hrs of infection in control. Hence, this compound could play a role as a therapeutic agent (Viszwapriya et al., 2016). Moreover, in *S. pyogenes*, 2,4-DBP effectively reduced biofilm formation, EPS production, and cell-surface hydrophobicity and restricted the initial adhesion of bacterial cells during biofilm formation. For bio-monitoring eco-toxicological studies, *C. elegans* is popularly used. As in Viszwapriya et al. (2016) who used *C. elegans* for assessing the toxicity of 2,4-DBP, they concluded the non-toxic nature of 2,4-DBP and considered it as apt for clinical applications. Apart from the present study, 2,4-DBP was found to be potent in constraining the biofilm formation besides considerably disrupting (*p* < 0.05) preformed biofilms in *C. albicans* (Padmavathi et al., 2015). In addition, 2,4-DBP, a known antioxidant, was investigated for its role in modulating the EPS in *Serratia marcescens* (Padmavathi et al., 2015). They reported a significant reduction in protein, polysaccharides, and eDNA components of EPS by *S. marcescens* when treated with 2,4-DBP, which would perhaps assist in biofilm disruption by facilitating the dissemination of antimicrobials into the biofilm. Recently in 2019, 2,4-DBP was reported to be isolated from *Bacillus licheniformis*, a thermophilic bacterium that thrives at 55°C the antibacterial activity against *S. aureus* and *P. aeruginosa* (Aissaoui et al., 2019). Hence, aforementioned studies support our results and provide evidence of 2,4-DBP as a potential candidate as a therapeutic agent. Besides the role of 2,4-DBP against bacteria and fungi, it is also reported to possess anticancer activity against human gastric adenocarcinoma AGS cells (Song et al., 2018).

Though LasR sits at the top of the *P. aeruginosa* QS hierarchy, *rhl* and *pqs* signaling regulons only partially overlap with *las* (Lee and Zhang, 2015). Therefore, inactivation or inhibition of the LasIR QS system may partially impair the Rhl and PQS signaling systems. There is considerable evidence that the Rhl signaling system negatively regulates the type-III secretion system (Hogardt et al., 2004; Bleves et al., 2005). Furthermore, previous reports also suggest that virulence of *P. aeruginosa* remains active in the Δ*lasR*-Δ*rhlR* double mutant, possibly due to the secretion of the effectors ExoT and ExoS by the type-III secretion system (Soto-Aceves et al., 2019). In our study, we observed that, though the treatment of 2,4-DBP reduces the QS gene expressions by 30–40%, the organism was still non-virulent when cocultured with the host cells (Figure 5). This suggests that, though this reduction in QS genes is not huge, it is sufficient to reduce the production of the virulence factors by 50–70%, which is definitely profound, and to possibly keep the *rhl* system fairly active to inhibit the type-III secretion system, further inhibiting virulence. Additionally, 2,4-DBP offers a possibility of its use as combination therapy with antibiotics as obsolete as ampicillin against multidrug-resistant *P. aeruginosa*. While further studies are needed to validate this interesting dual property of 2,4-DBP, molecules with such properties can serve as valuable therapeutic options. In this respect, our study with 2,4-DBP could serve as a starting point for the identification of such molecules, which can cause all-round inhibition of virulence as well as help in killing the pathogen.

By only reducing QS using a quorum-sensing inhibitor, it may not be possible to treat *P. aeruginosa* infections. However, the best approach might be to attenuate quorum-sensing-mediated traits, such as virulence and biofilm formation, as well as to combine this with clinically relevant drugs that together with the host’s immune system can act simultaneously to clear the pathogen. The main idea of our study was to achieve this by the use of 2,4-DBP. The considerable effect of the combination of 2,4-DBP with an obsolete antibiotic such as ampicillin achieves this to a large extent. It should be noted that it is not the inhibition of QS that makes 2,4-DBP valuable. Its ability of all-round inhibition of *P. aeruginosa* biofilm formation, production of virulence factors, and killing of the pathogen in combination with ampicillin, in addition to inhibiting QS, makes it a potential combination therapeutic agent.

Further, approaches to QS intervention claim to attenuate bacterial virulence without specifically inhibiting bacterial growth, suggesting that the immune system can regulate the infections *in vivo*. Nevertheless, strong experimental evidence against the validity of most of these hypotheses has emerged in recent years for the QS inhibitor in García-Contreras (2016). Moreover, many researchers believe that there are several challenges and limitations in anti-QS therapies that highlight three major properties attributed to QS inhibitors (Krzyżek, 2019). In order to develop truly solid QS inhibitor therapeutic alternatives to combat this remarkable pathogen, a much better understanding of its virulence and actions during infections is necessary. Even though the laboratory results are promising, it is undeniable that there is the need of thorough understanding of the knowledge of the impact of QS inhibition on the pathogen fitness in more convincing circumstances, such as interactions with a host, the external environment, and complex microbial communities (Liu et al., 2018).

To summarize, studies on anti-QS compounds/extracts from fungal sources are very few and are of recent origins. In fact, this has become a handicap for us to compare our results with other studies involving fungal extracts. Hence, we were forced to compare anti-QS activities of extracts/compounds of bacterial/plant origin. Nevertheless, it shows that the natural products are still the largest reservoir of compounds/metabolites for a range of ailments and for therapeutic use with fungi falling in line in the anti-QS realm also. To that extent, the present study revealed a promising role for selected fungi, isolated as endophytes from *T. procumbens*, after an initial screening. Also, we could isolate a pure compound 2,4-DBP from one of these fungi and demonstrate its potential as an anti-QS compound through various assays and experiments.

## Data Availability Statement

The datasets generated for this study can be found in the Genbank, KX987249.

## Author Contributions

RM and JK contributed to the conception, design, and experiment of the study. MK performed qRT-PCR and cell culture studies. CM performed the *in silico* studies under supervision of AM. RM wrote the first draft of the manuscript. SP, MK, CM, and AD wrote the sections of the manuscript. VV supervised the study. All authors contributed to the manuscript revision, and read and approved the submitted version.

## Conflict of Interest

The authors declare that the research was conducted in the absence of any commercial or financial relationships that could be construed as a potential conflict of interest.

## Abbreviations

2,4-DBP: 2,4-Di-tert-butylphenol
BAC: baicalein
MIC: minimum inhibitory concentration
QS: quorum sensing.
