# Supplementary material for: 2,4-Di-Tert-Butylphenol Isolated From an Endophytic Fungus, Daldinia eschscholtzii, Reduces Virulence and Quorum Sensing in Pseudomonas aeruginosa
Source: Front Microbiol. 2020 Jul 27;11:1668. doi: 10.3389/fmicb.2020.01668 (PMC7418596; doi:10.3389/fmicb.2020.01668)
Supplement: Supplementary file 1 [file Data_Sheet_1.PDF]

Supplementary Figures

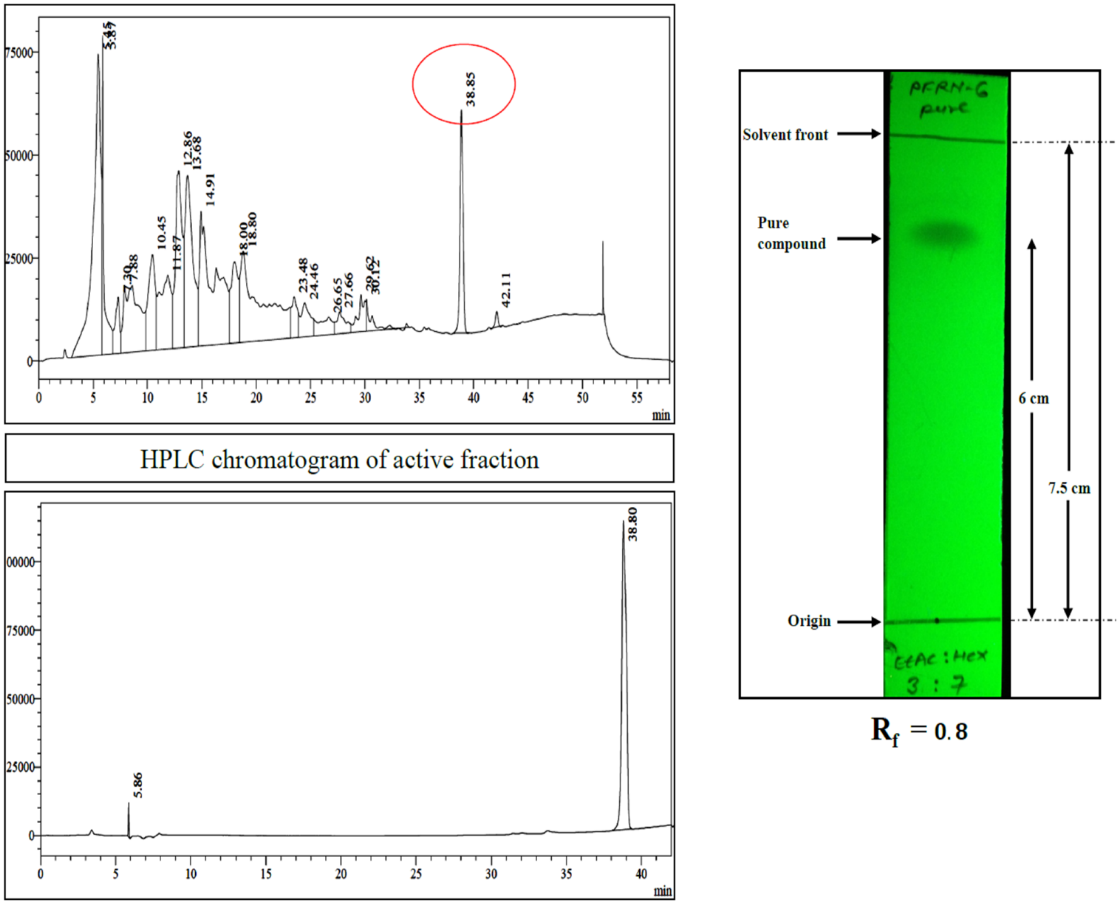

Figure S1. HPLC chromatogram of active fraction and purified compound. TLC and R_f value of purified compound at 254 nm are also shown.

A.

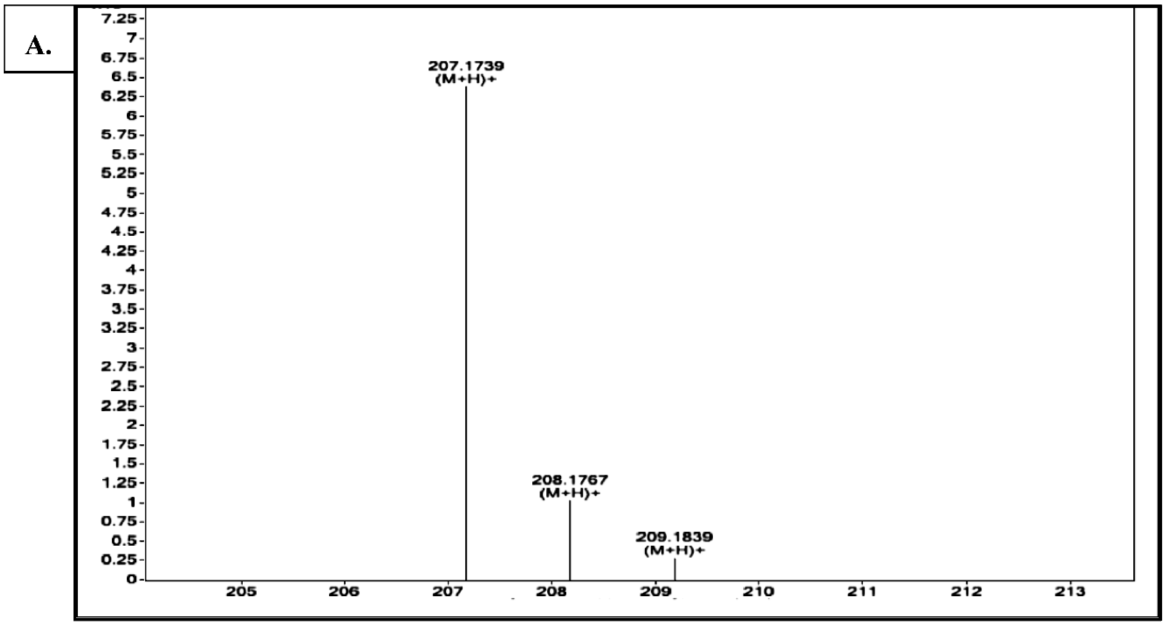

B.

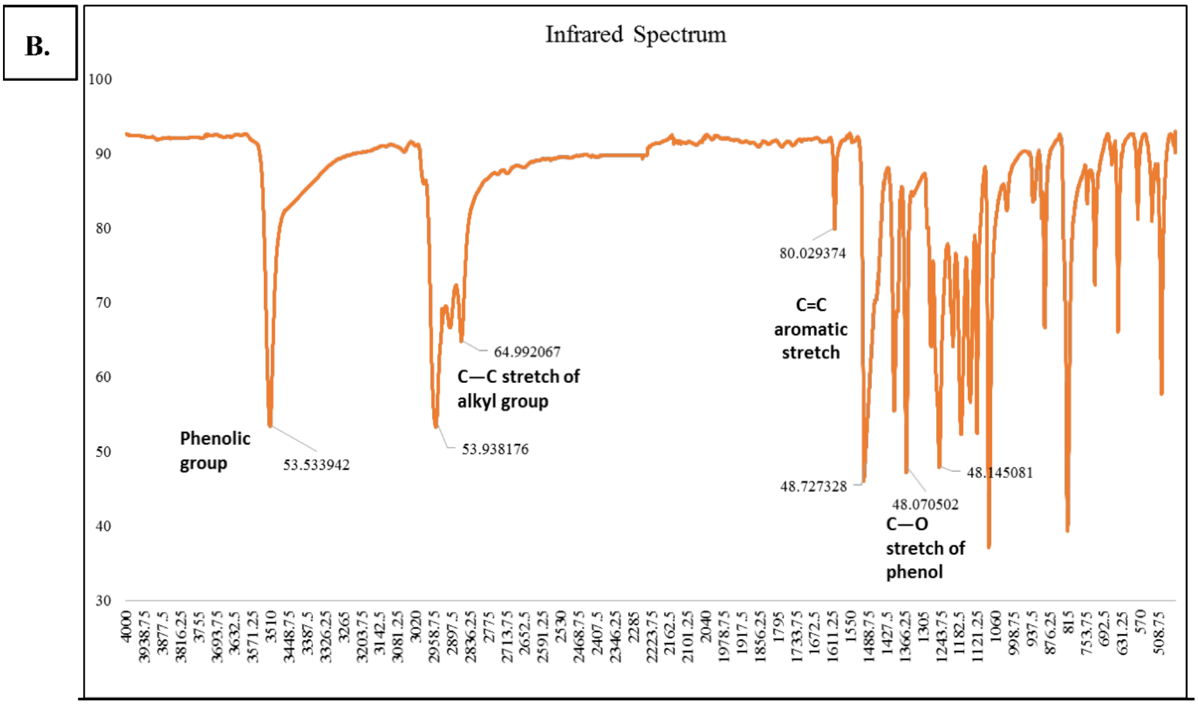

Figure S2. A) High-resolution mass spectrometry: The molecular mass of the compound detected by HRMS [HR-ESI (+)-MS] at m/z 206.1739 at positive mode [M+H]. B) Fourier-transform infrared spectroscopy (FTIR): FTIR spectrum of the compound.

A. ^{1}H NMR spectrum of compound

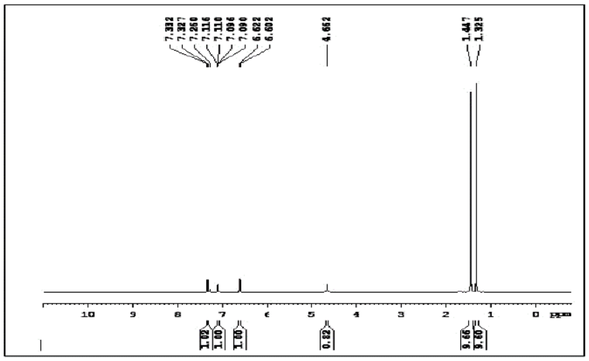

B. ^{13}C NMR spectrum of compound

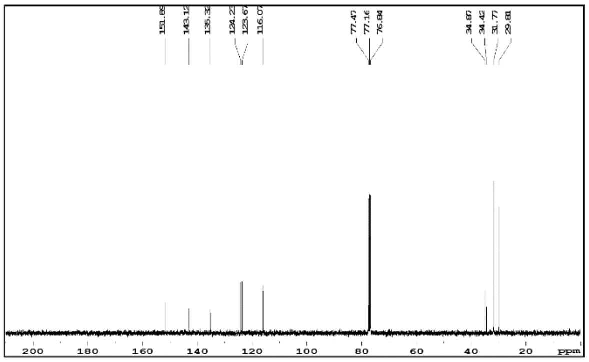

Figure S3. Structural elucidation of 2,4-DBP with the help of NMR spectra. Compound name - 2,4 - di-tert-butyl phenol. Molecular formula - $C_{14}H_{22}O$.

Supplementary Table 1

	Fungal Extract	Anti Quorum Sensing			
		Pseudomonas aeruginosa		*Chromobacterium violaceum*	
		250µg	500µg	250µg	500µg
1	TP4-6	-	14	16	19
2	TP4-4	-	14	14	18
3	TP4-2	-	-	-	13
4	TP4-6	-	-	-	20
5	TP3-8	14	16	-	15
6	TP3-1	-	14	-	16
7	TP3-2	16	17	-	16
8	TP3-3	13	15	-	--
9	TP3-4	-	-	-	14
10	TP3-5	15	16	-	14
11	TP3-6	-	15	-	17
12	TP4-8	14	14	14	18
13	TP4-3	14	16	-	17
14	TP4-7	-	14	-	16
15	TP4-12	-	13	-	16
16	TP4-10	13	14	14	20
17	TP4-1	14	17	14	16
18	TP2-1	11	12	10	13
19	TP4-9	14	16	16	19
20	TP2-3	14	16	15	18
21	TP2-6	18	20	21	23
22	TP4-5	14	16	15	17
23	TPR-2	-	13	-	16
24	TPR-5	13	14	14	20
25	TPR-6	14	16	16	19
26	TPR-8	13	15	13	17
27	TPR-9	14	16	16	19
28	TPR-1	-	-	-	13
29	TPR-3	-	-	-	20
30	TPR-4	-	17	-	17
31	TP3-6	-	15	-	17
32	TP3-7	-	14	15	20

Supplementary Table 2. List of Reverse transcriptase primers used in the study.

Gene	Primer	Sequence of RT primers
**LasI**	FP	GGCTGGGACGTTAGTGTCAT
	RP	AAAACCTGGGCTTCAGGAGT
**LasR**	FP	AAGGACAGCCAGGACTACGA
	RP	GTAGATGGACGGTTCCCAGA
**RhlI**	FP	TACCGGCATCAGGTCTTCAT
	RP	GATGGTCGAACTGGTCGAAT
**RhlR**	FP	CTGGGCTTCGATTACTACGC
	RP	CCCGTAGTTCTGCATCTGGT
**RpoD**	FP	CGAGACGATCAACAAGCTCA
	RP	GGCGATCTTCAGTACCTTGC

Supplementary Table 3. Docking result details of the cognate signaling molecule 3-oxo-C12-HSL (inducer), Baicalein, 2,4-DBP with LasR and RhlR.

Complex	Dock score	H-Bond Residues	H-Bond Distance (Å)	Binding energy	Hydrophobic interactions
**LasR-Inducer**	-4.28	NH1 TRP 60- O2 UNK<br>OG1 THR 75- N1 UNK<br>OD1 ASP 73- N1 UNK	3.01<br>3.30<br>3.00	-42.2	LEU 110, PHE 101, TYR 93, ALA 105, TRP 88, TYR 56, SER 129, TYR 64, LEU 36, ALA 127, TYR 47, ALA 50, VAL 57, ILE 52
**RhlR-Inducer**	-8.116	OE1 GLN 103- N4 UNK	3.17	-33.596	TRP 68, TYR 72, ASP 81, PRO 82, ALA 83, TRP 96, SER 97, ASP 98, ASP 102, LEU 107
**LasR-Baicalein**	-9.284	OH TYR 56- O3 UNK<br>NH2 ARG 61- O5 UNK	3.29<br>2.90	-65.425	LEU 36, GLY 38, LEU 39, LEU 40, TYR 56, ARG 61, TYR 64, ASP 73, THR 75, VAL 76, THR 115, LEU 125, GLY 126, ALA 127, SER 129
**RhlR-Baicalein**	-9.080	OH TYR 64- O4 UNK	3.00	-54.215	TRP 68, TYR 72, ASP 81, PRO 82, ALA 83, TRP 96, SER 97, ASP 98, LEU 100, ASP 102, GLN 103, LEU 107, ALA 111, LEU 116, SER 135
**LasR-2,4-DBP**	-8.457	NH2 ARG 61- O1 UNK	3.28	-2.970	LEU 36, GLY 38, TYR 47, ALA 50, TYR 56, ARG 61, TYR 64, ASP 73, VAL 76, ALA 127, SER 129
**RhlR-2,4-DBP**	-9.559	O TRP 68 - O1 UNK	2.75	-21.498	TYR 64, LEU 69, TYR 72, ASP 81, TRP 96, LEU 107, ALA 111
